# Baseline Mutations and Up-Regulation of PI3K-AKT Pathway Serve as Potential Indicators of Lack of Response to Neoadjuvant Chemotherapy in Stage II/III Breast Cancer

**DOI:** 10.3389/fonc.2021.784985

**Published:** 2022-04-04

**Authors:** Menghao Dong, Benjie Shan, Xinghua Han, Xiaotian Zhao, Fufeng Wang, Liuqing Zhu, Qiuxiang Ou, Xiaopeng Ma, Yueyin Pan

**Affiliations:** ^1^Department of Medical Oncology, The First Affiliated Hospital of University of Science and Technology of China (USTC), Division of Life Sciences and Medicine, University of Science and Technology of China, Hefei, China; ^2^Geneseeq Research Institute, Nanjing Geneseeq Technology Inc., Nanjing, China; ^3^Department of Thyroid and Breast Surgery, The First Affiliated Hospital of University of Science and Technology of China (USTC), Division of Life Sciences and Medicine, University of Science and Technology of China, Hefei, China

**Keywords:** neoadjuvant chemotherapy (NAC), pathologic complete response (pCR), *PIK3CA* mutations, PI3K-AKT pathway, stage II/III breast cancer (BC)

## Abstract

**Background:**

Neoadjuvant chemotherapy (NAC) has been expanded to hormone receptor (HR) positive breast cancer (BC) patients with operable disease, to increase the likelihood of breast-conserving surgery. Genomic profiling at baseline would reveal NAC response relevant genomic features and signaling pathways, guiding clinical NAC utilization based on patients’ genomic characteristics.

**Methods:**

We prospectively studied stage II/III BC patients who were eligible for breast-conserving surgery. Patients received epirubicin and cyclophosphamide for 4 cycles, followed by another 4-cycle docetaxel, and human epidermal growth factor receptor (HER2) positive patients were additionally treated with herceptin when using docetaxel (EC-T(H)). NAC responses were evaluated as pathologic complete response (pCR) or non-pathologic complete response (non-pCR). Genomic features related to NAC responses were identified by profiling baseline tumor tissues sampled one day before NAC, using whole-exome sequencing. Differentially expressed genes and up-/down-regulated pathways were investigated by performing RNA-sequencing.

**Results:**

A total of 25 stage II/III BC patients were enrolled, including 5 patients ultimately evaluated as pCR and 20 patients evaluated as non-pCR. *PIK3CA* (48%) and *TP53* (40%) mutations were enriched in patients not achieving pCR. Mutated phosphatidylinositol-3-kinase-AKT (PI3K-AKT) pathway and homologous recombinational repair pathway were also more frequently observed in patients evaluated as non-pCR. Significant arm-level amplifications (8q24.23 and 17q12) and deletions (1p32.2, 4p14, 7q11.23, 10q21.3, 11q23.3, etc.) were identified among patients not achieving pCR, while patients achieving pCR displayed no significant copy number alterations. Significantly up-regulated expression of PI3K-AKT pathway genes was also detected among patients failed to achieve pCR, compared to patients achieving pCR.

**Conclusion:**

Compared to BC patients achieving pCR to NAC, aberrant activation of PI3K-AKT pathway genes were more frequently observed in patients not achieving pCR, consistent with the significant up-regulation of PI3K-AKT pathway gene expression in the non-pCR subgroup. Together, these findings indicate that upregulated PI3K-AKT pathway serves as a potential indicator of lack of response to NAC in stage II/III BC patients, and other effective therapeutic options are urgently needed for those resistant patients.

## Introduction

Breast cancer (BC) is the most common cancer among females, and its mortality rate is also the highest for female cancer patients worldwide (1). Neoadjuvant chemotherapy (NAC) has become a widely used systemic therapy before definitive surgery, as it could reduce the extent of surgery, control the tumor size, and increase the probability of curability in early-stage BC (2, 3). For triple-negative breast cancer (TNBC) patients, it was suggested that anthracycline/taxane-based NAC should be offered if patients were node-positive and/or at least T1c. Similarly, anthracycline/taxane-based regimen in combination with trastuzumab were recommended to human epidermal growth factor receptor (HER2) positive BC patients with node-positive or high-risk node-negative (4). Although no evidence directly indicated the optimal NAC regimen in hormone receptor (HR)-positive/HER2-negative BC patients, NAC was suggested to be used instead of adjuvant chemotherapy, as patients treated with NAC had similar prognosis as those with adjuvant chemotherapy (5).

The most common endpoint of NAC is pathologic complete response (pCR), usually defined as “no invasive cancer and *in situ* cancer in the breast and axillary nodes” or “no invasive cancer in the breast and axillary nodes, irrespective of ductal carcinoma *in situ*” or “absence of invasive cancer in the breast irrespective of ductal carcinoma *in situ* or nodal involvement”. pCR can serve as a surrogate endpoint for the prognosis of disease-free survival and overall survival (6). Compared to the patients with non-pathologic complete response (non-pCR), patients achieving pCR had significantly superior disease-free survival, especially for HER2-enriched and TNBC patients (7).

Previous studies indicated that clinical characteristics and some biomarkers were related with pCR. Higher pCR percentages were observed among HR–/HER2+ (38.9%, 95% CI 33.2-44.9%) and TNBC (38.9%, 95% CI 33.2-44.9%) subtypes, compared to HR+/HER2– and HR+/HER2+ (8). Rates of pCR were also higher among patients with family BC history or carrying *BRCA1/2* mutations (9, 10). Additionally, higher ratios of CD8^+^ to forkhead box protein 3^+^ tumor infiltrating lymphocytes were detected in non-pCR TNBC patients with lower residual cancer burden (RCB) after NAC, representing that anti-tumor immune response was related with the response to NAC (11). However, genomic profiles do not act as a routine guideline for NAC regimen in BC, as the association of BC-related genes or signaling pathways with the response to NAC has not been comprehensively investigated (4).

Whole-exome sequencing (WES), covering almost all protein coding regions in the human genome, has previously been used to identify BC susceptibility genes and biomarkers of treatment response (12, 13). RNA sequencing (RNA-Seq), using next-generation sequencing (NGS) technology, can reveal the quantity of RNA, and detect differentially expressed genes (DEGs) by performing statistical analyses (14). DEGs in specific subgroup will be classified as up- or down-regulated genes, compared to a reference subgroup. Biological processes or signaling pathways associated with DEGs can further be identified (15).

In this study, we aimed to explore genomic features associated with NAC response among stage II/III BC patients who were eligible for breast-conserving surgery. Patients’ tumor tissue samples were performed with WES profiling before receiving epirubicin, cyclophosphamide, followed by docetaxel with or without herceptin (EC-T(H)). We then investigated DEGs between patients achieving and not achieving pCR, and identified up-/down-regulated pathways by RNA-Seq. Our results provided insights into baseline genomic indicators and key signaling pathways of NAC responses, and our findings could potentially help guide clinical NAC utilization.

## Methods

### Participants and Neoadjuvant Chemotherapy

Female patients with stage II/III primary BC, eligible for breast-conserving surgery, and suitable for receiving NAC were included in this study. All patients received EC-T (H): epirubicin (100 mg/m^2^), and cyclophosphamide (600 mg/m^2^) intravenous drip every 3 weeks for 4 cycles, followed by docetaxel (100 mg/m^2^) intravenously every 3 weeks for 4 cycles with or without herceptin. HER2+ patients were additionally treated with herceptin (8 mg/kg at first infusion, and then 6 mg/kg) every 3 weeks for 4 cycles when receiving docetaxel. Key exclusion criteria included receiving other BC treatment at the same time (e.g., neoadjuvant endocrine therapy and traditional Chinese medicine treatment), with NAC regimen changed depending on physicians’ decisions, no response records of NAC, or unavailable baseline tumor biopsies sampled one day before the beginning of NAC. Each participant provided informed written consent, and this study was approved by the ethics committees of Anhui Provincial Hospital (ethics number 2019KY086).

### Receptor Status Determination

The patient’s estrogen receptor (ER) and progesterone receptor (PR) status were determined by immunohistochemistry (IHC). The expression of ER and PR were quantified by the percent of stained cell nuclei. If the expression of each receptor was 1% or greater, the status of ER or PR was considered positive; otherwise, it was identified as negative status. Patients’ HER2 status were primarily analyzed by IHC, with scores of 0, 1+, 2+, or 3+. If the score was 0 or 1+, the status of HER2 was considered to be negative. HER2 positive samples included tumor samples with cores of 3+. Fluorescence *in situ* hybridization (FISH) was further performed on tumor tissues whose scores were 2+. In FISH analyses, a HER2 positive sample was identified if the ratio of HER2 to centromere 17 was over 2.0 (16).

### NAC Evaluation and Pathological Review

The response to NAC was primarily evaluated as pCR or non-pCR. After completing NAC, patients underwent surgical resection, and then specimens were sent to the pathology department for ascertaining surgical margin status. If residual invasive cancer was absent in the complete resected specimen and sampled regional lymph nodes, it was identified as pCR; otherwise, it was identified as non-pCR. NAC responses were further classified into 4 RCB categories: RCB-0 (pCR), RCB-1 (minimal residual disease), RCB-2 (moderate residual disease) and RCB-3 (extensive residual disease) (17).

### Baseline Sample Collection and Sequencing

The baseline tumor tissue, sampled one day before receiving NAC, underwent the complete procedure of formalin-fixed paraffin-embedded (FFPE) sample preparation before sequencing.

For WES, genomic DNA from the baseline FFPE sample was extracted with QIAamp DNA FFPE Tissue Kit (QIAGEN). Whole blood samples were used as normal controls, and genomic DNA from control samples was extracted with DNeasy Blood and Tissue Kit (QIAGEN). Extracted genomic DNAs were quantified by Qubit 3.0 using dsDNA HS Assay Kit (ThermoFisher Scientific). All samples were analyzed at a centralized clinical testing center (Nanjing Geneseeq Technology Inc.). Sequencing libraries were prepared using KAPA Hyper Prep Kit (KAPA Biosystems), and targeted enrichment was performed with the xGen Exome Research Panel and Hybridization and Wash Reagents Kit (Integrated DNA Technology). Enriched libraries were sequenced on Illumina Hiseq4000 platform using PE150 sequencing chemistry (Illumina), with a mean coverage depth of 150x for tumor tissue samples and 50x for matched normal blood control samples.

For RNA-seq, total RNA from FFPE samples was extracted with RNeasy FFPE Kit (QIAGEN) and quantified using Bioanalyzer 2100 (Agilent Technologies). Ribosomal RNA and residual genomic DNA were depleted by the KAPA Standard RNA-Seq Kit with RiboErase (HMR) and DNase digestion, followed by purification using the Agencourt RNA Clean XP Beads. Illumina compatible sequencing libraries were prepared using KAPA Stranded RNA-Seq Library Preparation Kit, including RNA fragmentation and priming, double-stranded cDNA synthesis, adaptor ligation and polymerase chain reaction amplification. Libraries were sequenced on Illumina Hiseq NGS platforms (Illumina).

### Mutation Calling and Sequencing Data Processing

For WES, FASTQ file quality was controlled by Trimmomatic, removing leading/trailing low quality (reading < 15) or N bases (18). High-quality reads were aligned to the reference human genome (hg19) using the Burrows-Wheeler Aligner Maximal Exact Match (BWA-MEM) with the default parameters (19). Local realignment around indels and base-quality score recalibration were applied using the Genome Analysis Toolkit (GATK) (20). Somatic single-nucleotide variants and small indels were called using MuTect and SCALPEL, respectively (21, 22). Somatic mutation and indel calls were further filtered by excluding: (1) alternate reads < 3 or variant allele frequency < 2%, (2) located in a non-protein-encoding region, or (3) the presence of one variant in public databases (Exome Variant Server, 1000 Genomes Project, and Exome Aggregation Consortium) at a population frequency > 1%. single-nucleotide variant and indel annotation was performed using ANNOVAR. Next, Somatic copy number alteration (CNA) analysis was performed by Genomic Identification of Significant Targets in Cancer (GISTIC), with the reference human genome of hg19. Aberrant regions with False Discovery Rates (q-values) below 0.2 were considered significant, and the most likely gene targets were identified within each significant region.

For RNA-seq, base calling was performed on bal2fastq (Illumina) to generate sequence reads in the format of FASTQ, followed by FASTQ file quality control using Trimmomatic (18). RNA-seq reads passing the quality control were then aligned to the reference human genome (hg19) using STAR algorithm (23).

### Differential Expression and Pathway Analysis

Differential expression analysis of RNA-seq data was performed between patients achieving and not achieving pCR, taking normalized read count data and discovering quantitative differences in expression levels, by DESeq2 based on negative binomial distribution. The significant DEG was considered if the adjusted p-value was less than 0.05 and absolute fold-change was greater than 2.

### Statistical Analysis

Fisher’s exact test was performed to compare the frequencies between subgroups. All quoted p-values of Fisher’s exact tests were two-tailed, and values less than 0.05 were considered to be statistically significant. Data were analyzed using R software (version 4.0.3).

## Results

### Patient Overview

**Figure 1:**
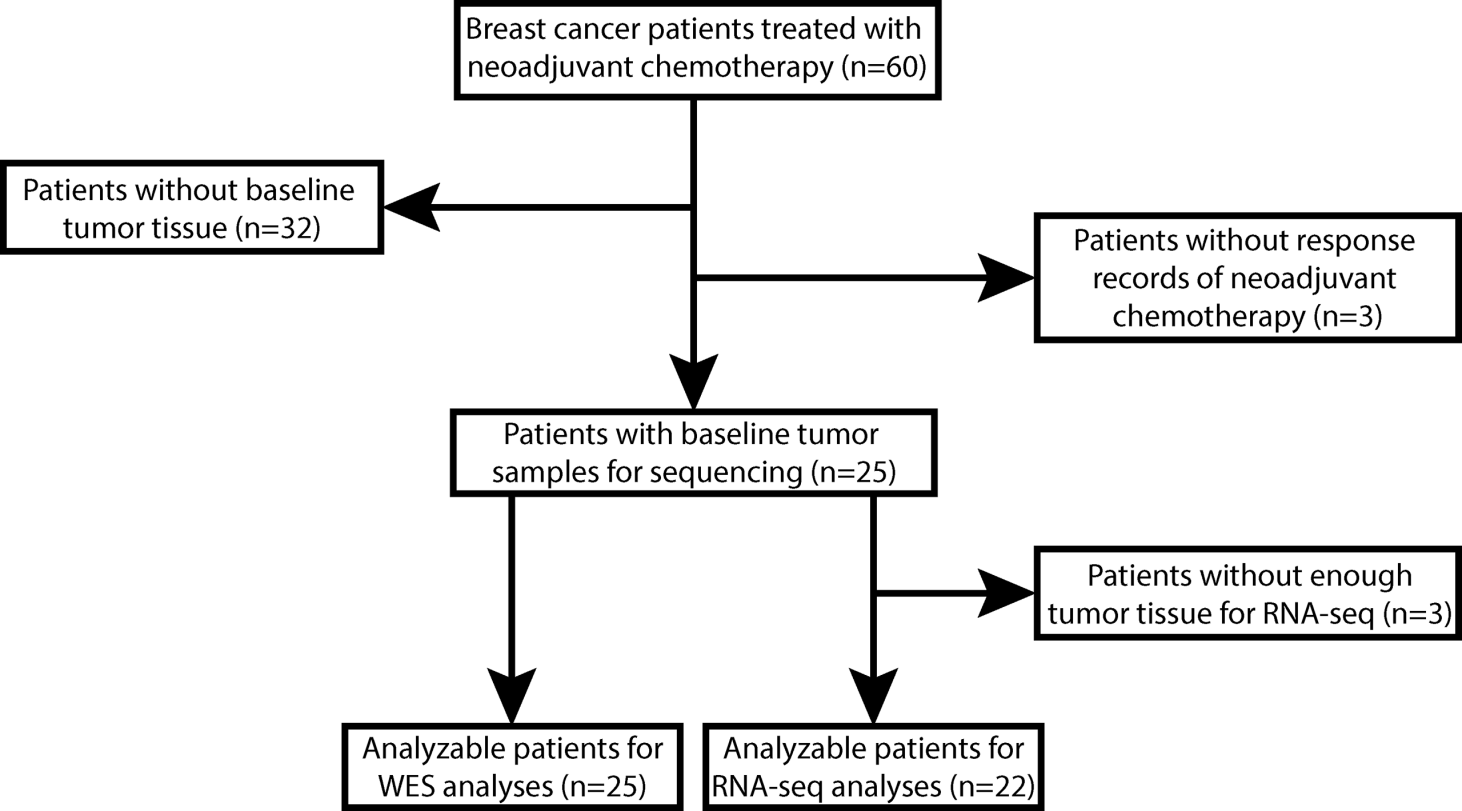
The flowchart of enrollment and analyzable patients. A total of 60 stage II/III breast cancer (BC) patients planned to receive breast-conserving surgery and neoadjuvant chemotherapy (NAC) of epirubicin, cyclophosphamide plus docetaxel with or without herceptin (EC-T(H)) were eligible for participation. We excluded 32 patients without available baseline tumor tissues and 3 patients without NAC response records, and the remaining 25 patients were enrolled in this study. All these 25 patients were analyzable for whole-exome sequencing (WES) data analyses, but 3 patients without enough tumor tissue for RNA sequencing (RNA-Seq) were excluded from RNA-Seq data analyses.

Between January 2019 and January 2021, 60 stage II/III primary BC patients planning to receive NAC before breast-conserving surgery in Anhui Provincial Hospital were eligible for participation. Thirty-five of them were excluded due to unavailable baseline tumor biopsy (n=32) or no response records of NAC (n=3). The remaining 25 patients were enrolled in this study, and their baseline tumor tissue samples were profiled using WES. However, 3 of 25 patients did not have enough tumor tissues for further RNA-seq. Therefore, 25 patients were included in the WES analyses, and 22 of them were included in the RNA-seq analyses (**Figure 1**).

The clinical characteristics of the 25 enrolled patients were summarized in **Table 1**. Five patients were evaluated as pCR after completing NAC, while the other 20 patients achieved non-pCR responses. Neither Luminal A nor HER2-enriched patients were evaluated as pCR. The median age at diagnosis of these 25 patients was 50 years (range, 28 to 69), and 13 (52%) patients were 50 years or older. At initial diagnosis, 18 of 25 (72%) patients were in clinical stage II, and 16 of 25 (64%) patients were classified as Liminal B subtype. No significant differences in clinical characteristics were detected between patients achieving and not achieving pCR, which was probably due to the limited sample size (**Table 1**).

**Table 1:** Overview of Patient Demographics and Clinical Characteristics.

Characteristic	Total (n = 25)	pCR (n = 5)	non-pCR (n = 20)	*p*-value^a^
Age at diagnosis, No. (%)				0.65
<50 y	12 (48)	3 (60)	9 (45)	
≥50 y	13 (52)	2 (40)	11 (55)	
Age at diagnosis, median (range), y	50 (28-69)	49 (28-59)	53 (32-69)	
Clinical stage at initial diagnosis, No. (%)				1.00
IIA	2 (8)	0 (0)	2 (10)	
IIB	16 (64)	4 (80)	12 (60)	
IIIA	5 (20)	1 (20)	4 (20)	
IIIC	2 (8)	0	2 (10)	
TNM stage at initial diagnosis, No. (%)				
T				1.00
1	3 (12)	0 (0)	3 (15)	
2	18 (72)	4 (80)	14 (70)	
3	3 (12)	1 (20)	2 (10)	
4	1 (4)	0 (0)	1 (5)	
N				1.00
1	20 (80)	5 (100)	15 (75)	
2	3 (12)	0 (0)	3 (15)	
3	2 (8)	0 (0)	2 (10)	
M				–
0	25 (100)	5 (100)	20 (100)	
Subtype at initial diagnosis, No. (%)				0.40
Luminal A	3 (12)	0 (0)	3 (15)	
Luminal B	16 (64)	3 (60)	13 (65)	
HER2-enriched	2 (8)	0 (0)	2 (10)	
Triple-negative breast cancer (TNBC)	4 (16)	2 (40)	2 (10)	
ER^b^				0.28
Positive (+)	17 (68)	2 (40)	15 (75)	
Negative (-)	8 (32)	3 (60)	5 (25)	
PR^b^				0.32
Positive (+)	12 (48)	1 (20)	11 (55)	
Negative (-)	13 (52)	4 (80)	9 (45)	
HER2^b^				
Positive (+)	6 (24)	1 (20)	5 (25)	1.00
Negative (-)	19 (76)	4 (80)	15 (75)	

pCR, pathologic complete response; non-pCR, non-pathologic complete response; ER, estrogen receptor; PR, progesterone receptor; HER2, human epidermal growth factor receptor 2.

a p-value calculated by Fisher’s Exact Test.

b Tested by immunohistochemistry (IHC) or fluorescence in situ hybridization (FISH).

### Baseline Somatic Mutations and CNAs Associated With Responses to NAC

**Figure 2:**
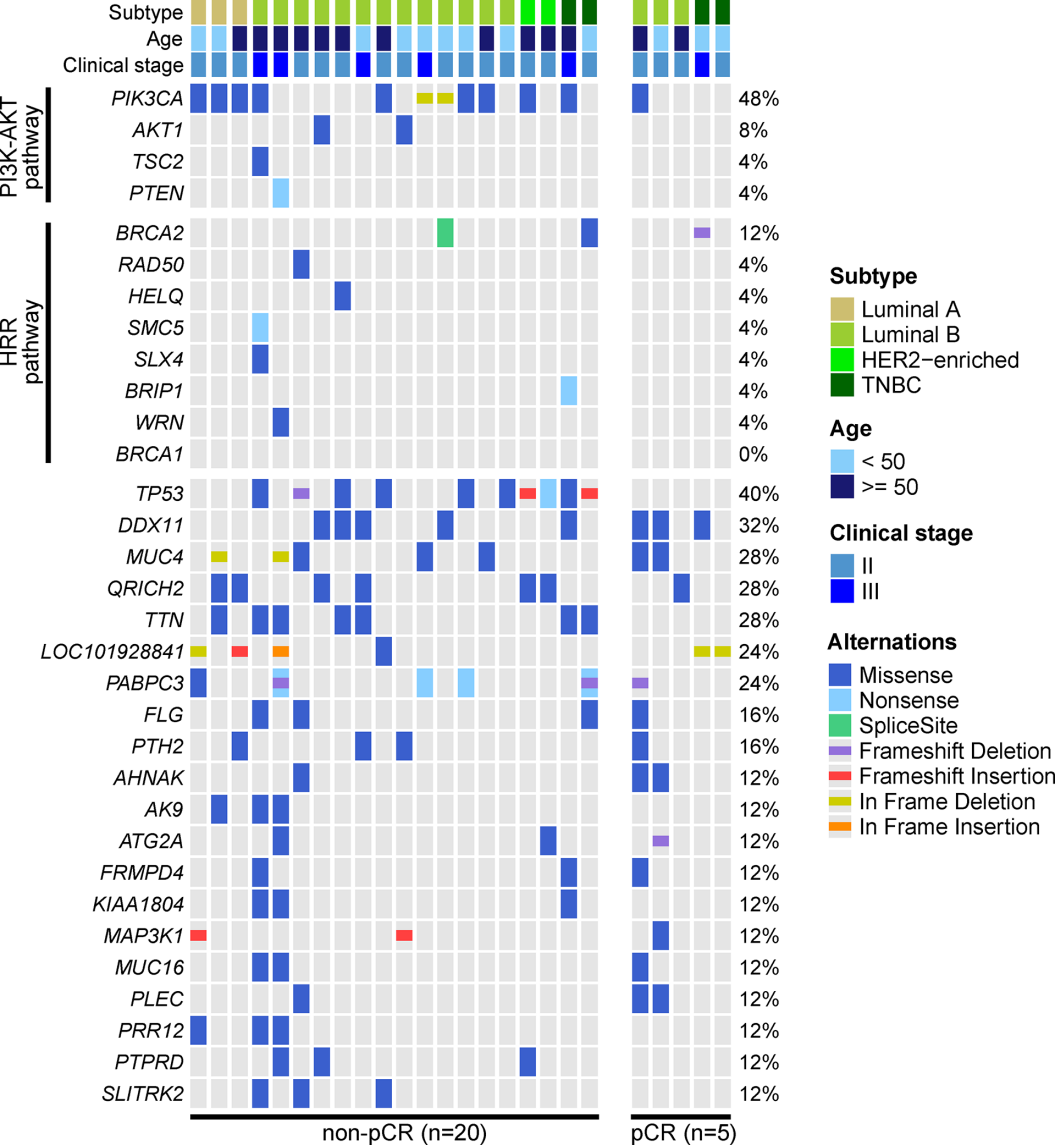
Genetic profile of baseline tumor tissue samples. The baseline tumor tissue samples of 25 patients were performed by WES. Altered genes of the phosphatidylinositol-3-kinase-AKT (PI3K-AKT) pathway and homologous recombinational repair (HRR) pathway and altered genes over 10% frequency were displayed. The most frequently altered genes were *PIK3CA*, *TP53*, *DDX11*, *MUC4*, *QRICH2*, and *TTN*. *PIK3CA* and *TP53* mutations were enriched in patients not achieving pCR, and mutated PI3K-AKT pathway and HRR pathway were also more frequently observed in patients not achieving pCR.

We performed WES on baseline tumor tissue samples of 25 enrolled patients. The top-three mutated genes were *PIK3CA* (48%), *TP53* (40%) and *DDX11* (32%). Only 1 of 12 *PIK3CA* mutations was observed in the 5 patients evaluated as pCR, and no *TP53* mutations were identified in these 5 patients. Although some mutated genes were exclusively detected in the non-pCR subgroup (e.g., *AK9*, *PRR12*, *PTPRD*, *SLITRK2*, etc.), their frequencies were relatively low. Additionally, baseline somatic mutations in 2 key BC-related signaling pathways, the phosphatidylinositol-3-kinase-AKT (PI3K-AKT) pathway and the homologous recombinational repair (HRR) pathway, were more likely to be observed in patients not achieving pCR (**Figure 2**).

The association between baseline mutations and responses to NAC was explored. *ZNF716* (*p*=0.03) and *RBMXL2* (*p*=0.03) were statistically associated with NAC responses, but extremely low frequencies (n=2) of these 2 mutated genes were observed in patients achieving pCR. We also observed a trend of *TP53* and *PIK3CA* mutations being enriched in patients not achieving pCR, while the associations were not statistically significant (*TP53*, *p*=0.06; *PIK3CA*, *p*=0.32). Similarly, neither PI3K-AKT (*p*=0.12) nor HRR pathway (*p*=0.48) showed statistically significant relationship with NAC responses (**Table 2**). The baseline somatic mutation of each RCB category and BC subtype were summarized in **Tables 3** and **4**, respectively. Although *TP53* mutations appeared to be enriched in patients not achieving pCR, no patients in RCB Index 2 were detected as *TP53* mutation positive. Mutated PI3K-AKT pathway were more frequently observed in RCB Index 2 or 3 patients than patients in the RCB Index 0 or 1 subgroup (**Table 3**).

**Table 2:** Genomic Features in Relation to Pathologic Complete Response (pCR).

Genomic Features	pCR, No.	non-pCR, No.	*p*-value^a^
**Genomic Mutation**			
*ZNF716*			**0.03**
Mutated	2	0	
Wild-type	3	20	
*RBMXL2*			**0.03**
Mutated	2	0	
Wild-type	3	20	
*TP53*			
Mutated	0	10	0.06
Wild-type	5	10	
*PIK3CA*			0.32
Mutated	1	11	
Wild-type	4	9	
**Signaling Pathway**			
PI3K-AKT			0.12
Mutated	1	14	
Wild-type	4	6	
HRR			0.48
Mutated	1	7	
Wild-type	4	13	

pCR, pathologic complete response; non-pCR, non-pathologic complete response; HRR, homologous recombinational repair.

a p-value calculated by Fisher’s Exact Test. p-values smaller than 0.05, representing statistically significant differences, are in bold.

**Table 3:** Genomic Features in Relation to Residual Cancer Burden (RCB) Index.

	RCB Index				
Features	0, No.	1, No.	2, No.	3, No.	*p*-value^a^
**Genomic Mutation**					
*ZNF716*					0.05
Mutated	2	0	0	0	
Wild-type	3	4	6	10	
*RBMXL2*					0.05
Mutated	2	0	0	0	
Wild-type	3	4	6	10	
*TP53*					**< 0.01**
Mutated	0	3	0	7	
Wild-type	5	1	6	3	
*PIK3CA*					0.33
Mutated	1	1	4	6	
Wild-type	4	3	2	4	
**Signaling Pathway**					
PI3K-AKT					**0.02**
Mutated	1	1	6	7	
Wild-type	4	3	0	3	
HRR					0.07
Mutated	1	1	0	6	
Wild-type	4	3	6	4	

pCR, pathologic complete response; non-pCR, non-pathologic complete response; RCB, residual cancer burden; HRR, homologous recombinational repair.

a p-value calculated by Fisher’s Exact Test.

p-values smaller than 0.05, representing statistically significant differences, are in bold.

**Table 4:** Genomic Features in Relation to Breast Cancer Subtypes.

Features	Luminal A, No.	Luminal B, No.	HER2-enriched, No.	TNBC, No.	*p*-value^a^
**Genomic Mutation**					
*PIK3CA*					0.24
Mutated	3	7	1	1	
Wild-type	0	9	1	3	
*TP53*					0.22
Mutated	0	6	2	2	
Wild-type	3	10	0	2	
*DDX11*					0.52
Mutated	0	6	0	2	
Wild-type	3	10	2	2	
*MUC4*					0.48
Mutated	1	6	0	0	
Wild-type	2	10	2	4	
*QRICH2*					**0.02**
Mutated	2	3	2	0	
Wild-type	1	13	0	4	
*TTN*					0.73
Mutated	1	4	0	2	
Wild-type	2	12	2	2	
*LOC101928841*					0.08
Mutated	2	2	0	2	
Wild-type	1	14	2	2	
*PABPC3*					1.00
Mutated	1	4	0	1	
Wild-type	2	12	2	3	
*ZNF716*					
Mutated	0	2	0	0	1.00
Wild-type	3	14	2	4	
*RBMXL2*					
Mutated	0	2	0	0	1.00
Wild-type	3	14	2	4	
**Signaling Pathway**					
PI3K-AKT					0.12
Mutated	3	10	1	1	
Wild-type	0	6	1	3	
HRR					1.00
Mutated	0	5	0	3	
Wild-type	3	11	2	1	

HER2, human epidermal growth factor receptor 2; TNBC, triple-negative breast cancer; HRR, homologous recombinational repair.

a p-value calculated by Fisher’s Exact Test.

p-values smaller than 0.05, representing statistically significant differences, are in bold.

**Figure 3:**
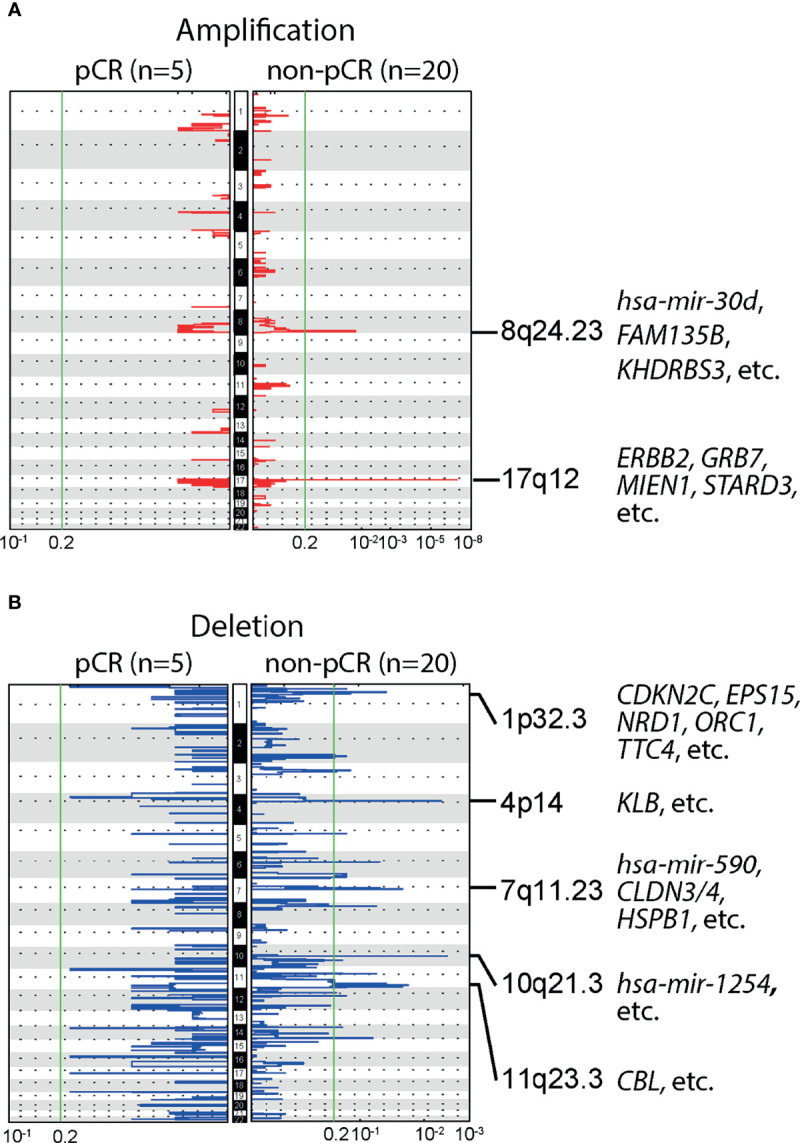
Baseline arm-level copy number alterations (CNAs) and responses to neoadjuvant chemotherapy (NAC). **(A)** Significant amplifications were detected in the chromosomal region 8q24.23 and 17q12, in patients not achieving pCR, but no significant amplifications were observed in patients achieving pCR. Of note, *ERBB2*, a gene closely related to breast cancer (BC) was located in 17q12. **(B)** Multiple significant deletions were detected in patients not achieving pCR, and five regions with the highest *q*-values were 1p32.3, 4p14, 7q11.23, 10q21.3,11q23.3.

We further studied the association of NAC responses with baseline CNAs at the chromosomal arm level. Many BC-related genes, such as *ERBB2*, *GRB7*, *MIEN1* and *STARD3*, were located in the region of chromosome 17q12, where significant amplification was observed among patients with non-pCR. Significant amplifications were also detected on chromosome 8q24.23, in the non-pCR subgroup (**Figure 3A**). Compared to amplifications, deletions were more common in patients not achieving pCR, such as 1p32.3, 4p14, 7q11.23, 10q21.3,11q23.3, etc., and BC relevant genes were frequently observed in the wide peak of each deletion region (**Figure 3B**). All baseline arm-level CNAs, with wide peak boundaries and genes in peaks, were shown in the **Supplementary Table**.

### Differential Expression and Up/Down-Regulated Pathways

**Figure 4:**
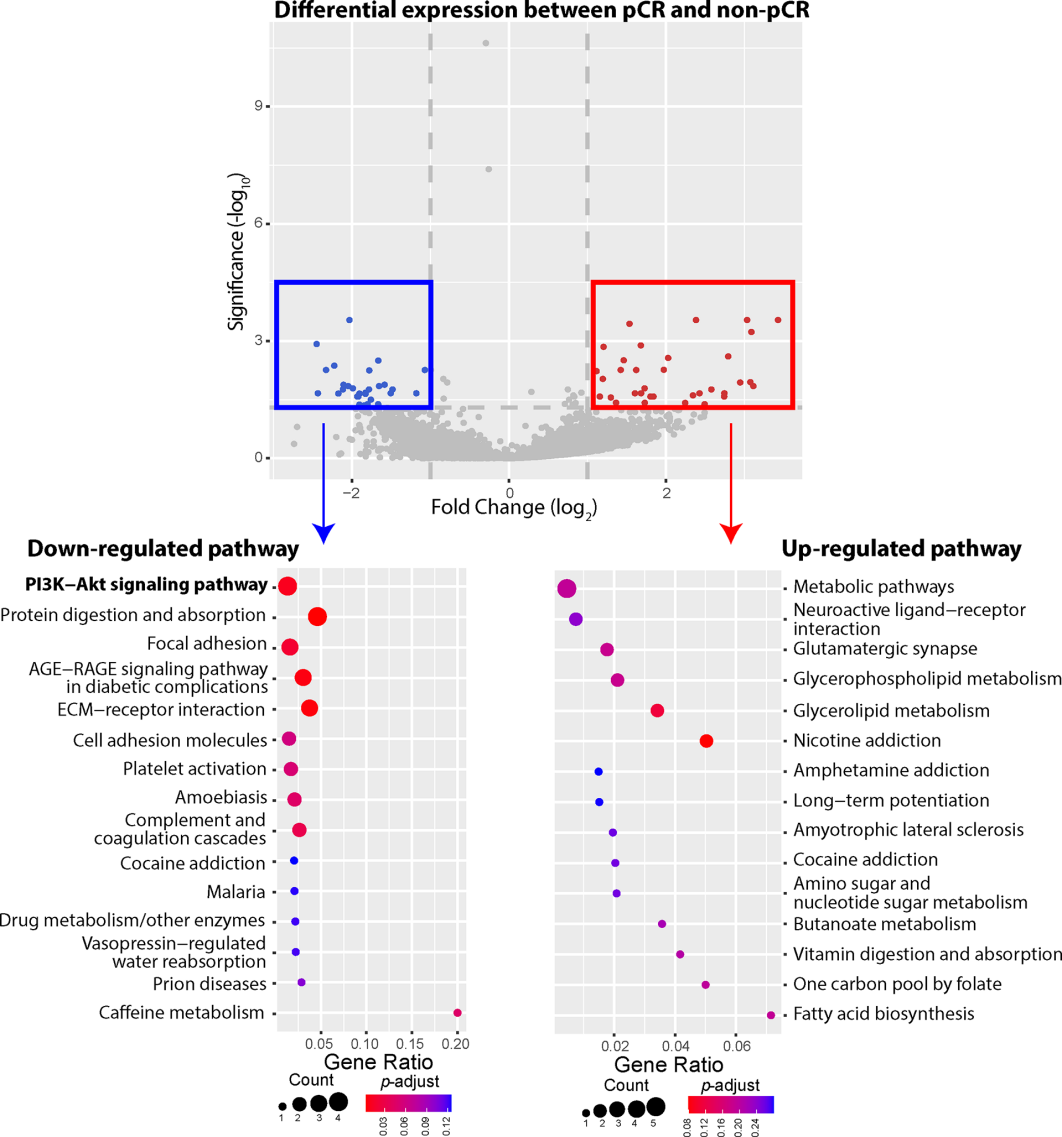
Differentially expressed genes (DEGs) and up-/down-regulated signaling pathways between pathologic complete response (pCR) patients or non-pathologic complete response (non-pCR) patients. A total of 61 DEGs were identified between the pCR subgroup and the non-pCR subgroup, including 34 genes expressed at higher levels and 27 genes expressed at lower levels, in the pCR subgroup, with absolute fold-change greater than 2. The phosphatidylinositol-3-kinase-AKT (PI3K-AKT) pathway was significantly down regulated among patients achieving pCR, with 4 lower expressed genes. Other down-regulated pathways included protein digestion and absorption, focal adhesion, AGE-RAGE, ECM-receptor, etc. On the other hand, higher expressed genes belonged to the pathways of metabolic pathways, neuroactive ligand-receptor interaction, glutamatergic synapse, glycerophospholipid metabolism, glycerolipid metabolism, nicotine addiction pathways, etc.

Our WES data showed that most observed mutations of *PIK3CA* gene in patients who failed to achieve pCR were gain-of-function mutations, including *PIK3CA*-H1047R, *PIK3CA*-E545K, *PIK3CA*-C420R, etc. We thus compared 3 patients achieving pCR with 19 patients not achieving pCR, investigating whether gain-of-function mutations of *PIK3CA* gene could influence the gene expression of PI3K-AKT signaling pathway (**Figure 4**). Compared to the patients who failed to achieve pCR after NAC, 27 DEGs involved in several down-regulated pathways were expressed at significantly lower levels among patients achieving pCR, including *COL1A1*, *COL1A2*, *COMP*, and *CREB3L1* genes belonging to PI3K-AKT pathway. In other words, patients not achieving pCR displayed up-regulated gene expression of PI3K-AKT pathway, among whom gain-of-function mutations of PI3K-AKT pathway were identified in previous WES analysis. On the other hand, 34 DEGs were expressed significantly higher among pCR patients, and the most frequently up-regulated pathways, such as metabolic pathways, neuroactive ligand-receptor interaction, glutamatergic synapse, etc., did not have strong relationship with BC development or lack of response of NAC.

## Discussion

This study reported WES and RNA-Seq data on baseline tumor tissue samples of patients who were treated with NAC of EC-T(H). We compared the genomic features of mutated genes and signaling pathways, DEGs and up-/down-regulated signaling pathways between patients achieving and not achieving pCR.

*PIK3CA* and *TP53* mutations were more frequently observed in patients not achieving pCR than patients achieving pCR, while this trend was not statistically significant due to the relatively small sample size. However, our findings of mutated *PIK3CA* genes aligned to a previous study including 851 BC patients. In that study, Loibi et al. reported a significantly higher pCR rate among non-carriers of *PIK3CA* mutation, especially HER2+ patients. An reduced response rate of nab-paclitaxel was also observed in BC patients harboring *PIK3CA* mutations (24). In another study of Loibi et al. after receiving taxane-based chemotherapy plus herceptin and/or lapatinib, a significantly lower pCR rate and worse disease-free survival were observed in HR+ patients with *PIK3CA* mutations (25). Additionally, in our study, of 11 patients with altered *PIK3CA* gene in the non-pCR subgroup, 5 patients harbored *PIK3CA*-H1047R mutation. GeparSixto, a prospective randomized phase II clinical trial including 92 TNBC patients treated with anthracycline-based neoadjuvant chemotherapy, reported a strong association between *PIK3CA*-H1047R mutation and a lower pCR rate (26). On the other hand, the association between *TP53* mutations and NAC response rate was not completely consistent across different studies. For instance, one study including 450 BC patients receiving anthracycline/taxane-based NAC indicated that *TP53* mutations were not statistically associated with pCR (27); however, a meta-analysis suggested that altered *TP53* gene was related to better response rates, and this association remained significant when BC patients were stratified according to their NAC regimens (28). Hence, further larger and well-designed prospective studies are required to evaluate the predictive role of *TP53* mutation in NAC of EC-T(H).

In our study, altered *RBMXL2* and *ZNF716* genes were significantly enriched in patients achieving pCR to the EC-T(H) regimen, whereas the frequencies of these two gene were extremely low. The association of *RBMXL2* gene, controlling for splicing patterns during meiosis and being essential for male fertility (29), with BC progression or NAC resistance was not well investigated by previous studies. Additionally, few studies have revealed the relationship between *ZNF716* and BC, even though another type of zinc finger protein could inhibit the proliferation of BC cells by regulating P53 stability (30). Thus, our findings on these two genes should be interpreted with caution, and further studies with large sample size are needed.

The 8q24.23 chromosomal region of patients not achieving pCR was amplified, in which several genes related to treatment outcomes of BC patients, such as *ST3GAL1* and *miR-30b*. Fan et al. reported that silencing *ST3GAL1* gene could reduce GDNF-mediated cell proliferation of BC cells *in vitro*, and *ST3GAL1* overexpression was associated with poor clinical outcomes in BC patients with late-stage disease (31). *miR-30b* was identified as a herceptin response regulator in another *in vitro* study on HER2+ BC cell lines, as the target gene of *miR-30b*, *CCNE2*, could probably contribute to herceptin resistance (32). Amplification was also detected in the 17q12 chromosomal region of patients with non-pCR responses, including the *ERBB2* gene which is related to BC development and progression. It was demonstrated that the amplification of *ERBB2* could affect the sensitivity of doxorubicin, another type of anthracyclines, to BC, which was consistent with our findings (33). *GRB7*, another gene playing an important role in BC development, was also located in this region. Previous study suggested that *GRB7* overexpression was an independent factor of poor prognosis (34). Furthermore, we observed several arm-level deletions of BC tumor suppressor genes and favorable BC prognostic genes in patients who failed to achieve pCR, including *CDKN2C* (35), *EPS15* (36), etc. Of note, there were a considerable number of genes related to BC development and progression in the regions where deletions were identified, including *CDKN2C*, *EPS15*, *CBL*, etc. Their associations with pCR rate of NAC should be further investigated separately.

Our WES data also demonstrated that *PIK3CA* mutations were the main mutations in PI3K-AKT signaling pathway, which comprises a group of intracellular signal transducer enzymes. This pathway was very likely to be associated to the response to NAC for BC patients. *PIK3CA* mutations and AKT activation by phosphorylation (pAKT) are frequently detected in BC, and pAKT regulates growth, proliferation, differentiation, tumorigenesis and other critical cellular activities (37, 38). BC tumor cells with *PIK3CA* mutations had lower probability of early apoptosis after being treated with epirubicin, and abnormal protein expression in PI3K-AKT signaling pathway, suggesting the association between chemotherapy resistance and activated PI3K-AKT pathway in TNBC (39). In one BC study, mutated PI3K-AKT pathways were detected among 29.8% TNBC patients, and over 70% of these patients carried *PIK3CA* mutations, which was consistent with our findings in this study including 4 BC subtypes (40). We further observed that the PI3K-AKT pathway was up-regulated among patients not achieving pCR, compared to those with pCR to NAC. Although there is no sufficient evidence supporting that mutated *PIK3CA* genes or mutated PI3K-AKT pathways are associated with treatment benefit of anthracyclines for BC, the interaction between mutated *PIK3CA* gene/PI3K-AKT pathway and chemotherapy agents, like anthracyclines and taxanes, has been indicated *in vitro* (41). For example, doxorubicin could induce AKT activation in BC cells, and overexpression of AKT in BC cells transfected with HER2 could result in the resistance to doxorubicin or other chemotherapeutic agents (42, 43). Additionally, lower pCR rates were observed in HER2+ BC patients with *PIK3CA* alterations than without *PIK3CA* alterations, even though the association between PI3K-AKT pathway biomarkers and docetaxel benefit was not identified (44–46). In our study, both epirubicin and docetaxel were included in the NAC regimen, and arm-level *ERBB2* amplification was of note detected within patients not achieving pCR. Thus, the combination of up-regulated PI3K-AKT pathway and *ERBB2* amplification could partially explain why some patients achieved relatively poor responses to NAC, in addition to other altered genes that we discussed before.

According to the National Comprehensive Cancer Network guidelines, the Oncotype DX breast recurrence score tests could help personalize systemic adjuvant treatment of HR+/HER2- and node-negative BC patients with tumor size greater than 0.5 cm. This genomic test is able to predict BC patients’ benefits of chemotherapy, by analyzing 21 cancer related genes and assigning a recurrence score (47). However, currently, genomic profiles are not strongly recommended as a routine guideline for clinical decisions on NAC, even though many studies have investigated whether genomic profiles could help BC patients select the optimal NAC regimen. A previous study suggested that chemoresistant genes existed prior to NAC, and clonal dynamics of these genes were involved in chemoresistance development (48). Another study demonstrated that BC patients with deficient *BRCA* mutations displayed higher pCR rates than *BRCA*-proficient or wild type patients, and the relationship remained significant in Luminal BC subtypes. Of note, no differences in survival were observed between patients with and without *BRCA* mutations (49). However, another research on taxane- and/or anthracycline-based NAC revealed that the *BRCA* alternation was associated with reduced progression-free survival in BC patients, in addition to poorer clinical response rates (50). Other BC related genetic aberrations predicting chemosensitivity included *TP53* mutation, *PIK3CA* mutation, *ERBB2* amplification, and *CCND1/2* amplification (51). Moreover, conducting genomic testing before NAC could also help define mutations predicting sensitivity of chemotherapy and survival in poor-prognosis BC patients, such as *MUC17* and *PCNX1* genes (52). Hence, we proposed that BC patients could take genomic sequencing for selecting optimal options of NAC and detecting potential NAC drug resistance.

It is of great clinical importance to explore more effective therapeutic options for patients who are resistant to the EC-T(H) NAC regimen. In general, extended adjuvant therapy, including chemotherapy, target therapy, and radiotherapy, may be an option for patients who fail to achieve pCR to EC-T(H). Similar to distinctive strength of associations between pCR and prognosis in each BC subtype (6), the development of subsequent treatment plans for BC patients with different subtypes varies considerably. The CREATE-X study suggested that patients not achieving pCR could consider the addition of capecitabine to improve the prognosis, especially TNBC patients, with 13.7% increase in 5-year disease-free survival rate (53). Endocrine therapy (ET) plus CDK4/6 inhibitors might be an option for HR+/HER2- patients, among whom significant improvement in prognosis was not observed in the CREATE-X study. In the monarchE trial, HR+/HER2- early BC patients receiving Abemaciclib for 2 years combined with ET had significantly superior invasive disease-free survival than those using ET alone (54). Hence, U.S. Food and Drug Administration has approved Abemaciclib with ET in the treatment of early BC. However, neither the PENELOPE-B trial nor the PALLAS trial showed HR+/HER2- patients could have improved invasive disease-free survival by receiving Palbociclib for 1 or 2 years combined with ET (55, 56). For HER2+ BC patients not achieving pCR, they could potentially receive T-DM1 in adjuvant therapy, with reduced free of invasive disease rate and distant recurrence rate by 11.3% and 6.7% in 3-year follow-up, respectively (57). Postmastectomy radiotherapy might be another option for patients with residual disease after NAC. Fowble et al. suggested that patients in clinical stage III could benefit from radiotherapy, regardless of their responses to previous NAC (58). Furthermore, BC patients with potential resistance to EC-T(H) NAC regimen might consider receiving other neoadjuvant therapy regimens to improve pCR rates. The GeparSixto trial suggested that the pCR rate increased by 16.3% when carboplatin was added into the anthracycline-based NAC regimen for TNBC patients, whereas the improvement of pCR rate was not significant in HER2+ BC patients (59). A meta-analysis including 9 randomized clinical trials also suggested higher a pCR rate in patients receiving platinum-based NAC, compared to those treated with platinum-free NAC; however, BC patients with *BRCA* mutations did not show significantly increased pCR rates when receiving platinum-based NAC (60). For patients harboring *PIK3CA* mutations, the addition of letrozole to neoadjuvant ET might help improve patients’ disease-free survival and overall survival (61). However, the NeoALTTO trial demonstrated that HER2+ patients with *PIK3CA* mutations did not had a pCR rate to neoadjuvant HER2-targeted therapy as high as patients with wide-type counterparts, and suggested the possibility of using anti-HER2 treatment combined with PI3K inhibitors (62). Compared to pan-PI3K inhibitors [such as Buparlisib and Pictilisib (63)], frequently added in ET or paclitaxel treatment for advanced BC patients, isoform-specific PI3K inhibitors [such as Alpelisib and Taselisib (63)] were generally used according to specific mutated genes in PI3K-AKT pathway. The addition of Taselisib to neoadjuvant ET was associated with higher proportion of objective response among HR+/HER2- stage I-III BC patients, but the improvement in pCR rate was not significant (64). Another study focused on HR+/HER2- BC patients with T1c-T3 disease showed that the combination of Alpelisib with 24-week neoadjuvant letrozole treatment could not significantly improve patients’ responses (65). Although our study revealed that mutated PI3K-AKT pathway could potentially indicate the lack of response to EC-T(H) regimen, further prospective studies or clinical trials should be launched to investigate how to personalize optimal treatment to BC patients with NAC resistance, according to patients’ receptor status and genomic profiles.

The main strength of this study was using WES and RNA-Seq simultaneously to explore genomic characteristics related to the NAC response for BC patients. The association of PI3K-AKT pathway with NAC responses was primarily investigated by somatic mutations in baseline tumor tissues and then confirmed by pathway analyses based on RNA-Seq data. Higher-expressed genes and up-regulated PI3K-AKT pathway in patients not achieving pCR could probably explain how mutated PI3K-AKT pathway genes, such as *PIK3CA*, *AKT1* and *PTEN*, influence the response to the NAC of EC-T(H). Another strength was the time point of sampling baseline tumor tissues. Compared to other retrospective studies where baseline tumor tissues were sampled at initial diagnoses or couples of weeks before NAC, our baseline tumor tissue biopsies were sampled one day before the beginning of NAC. This means we did not need to consider the confounding effects of mutation changes, induced by unknown treatments between diagnosis and NAC.

On the other hand, the primary limitation of our study was the relatively small sample size. Although the EC-T(H) was one of top recommended NAC regimens, we had to exclude patients who could and did receive other potentially beneficial and feasible BC treatment concurrently with NAC of EC-T(H). In addition, our patients were counselled to avoid using non-evidence-based treatment or other treatment without healthcare professionals’ authorization, whereas patients’ compliance to the EC-T(H) regimen was not as good as expected, leading to a part of potentially eligible participants being further excluded. As a result, we observed a series of trends without statistical significance, such as the enrichment of mutated *PIK3CA* and *TP53* genes in patients not achieving pCR compared to patients with non-pCR, and the potential relationship between mutated PI3K-AKT pathway and non-pCR. Therefore, in the next phase, multi-center research with a larger sample size is needed. Another limitation of our study was that both HER2+ patients with herceptin treatment and HER2- patients not receiving herceptin were included in this study. However, due to the limited sample size, we were not able to perform subgroup analyses in HER2+ or HER2- patients separately, to further explore the potential association between mutated PI3K-AKT pathway and lack of response to NAC in each subpopulation. In our data, a total of 6 patients with positive HER2 status, including 4 Luminal B and 2 HER2-enriched patients, were treated with herceptin, and 1 of 6 successfully achieved pCR to EC-T(H). Hence, further studies focused on HER2+ BC patients receiving NAC of EC-T(H) could be launched in the future. For instance, comparing the strength of association of mutated PI3K-AKT pathway with lacking response to the EC-T(H) NAC regimen, between Luminal B patients with and without positive HER2 status. Finally, the tumor tissue biopsies were collected for sequencing in our study, but tissue samples might not comprehensively capture the entire genomic profile of the tumor due to the heterogenicity of BC (54).

## Conclusion

In summary, our data suggest that patient’s baseline genomic profile could be taken into consideration when making NAC regimen decisions for stage II/III BC patients. Higher aberrancy of PI3K-AKT pathway genes and up-regulated gene expression were identified in baseline tumor biopsies of patients not achieving pCR to EC-T(H) regimen, as compared to patients achieving pCR. Our WES data also revealed that patients not achieving pCR had significant arm-level CNAs of breast cancer-relevant genes, including *ERBB2* amplification. Together, these findings indicate that upregulated PI3K-AKT signaling pathway serves as potential indicator of lack of response to NAC in stage II/III BC patients, and other effective therapeutic options are urgently needed for those resistant patients.

## Data Availability Statement

The original contributions presented in the study are publicly available. This data can be found here: https://ngdc.cncb.ac.cn/gsa-human/, HRA002168. Further inquiries can be directed to the corresponding author/s.

## Ethics Statement

The studies involving human participants were reviewed and approved by The Ethics Committees of Anhui Provincial Hospital (Ethics Number 2019KY086). The patients/participants provided their written informed consent to participate in this study.

## Author Contributions

MD and BS contributed equally to the work. YP designed the study. MD was responsible for patient recruitment and registration. BS and XH collected samples and data. XM and YP provided administrative support. XZ, FW, LZ, and QO analyzed data and interpreted results. All authors wrote and reviewed the manuscript. All authors contributed to the article and approved the submitted version.

## Funding

This investigation was supported by University of Science and Technology of China (New Medicine Joint Foundation of USTC, grant No. WK9110000058), Anhui Provincial Department of Science and Technology (Anhui Science and Technology Research Project, 1704a0802148; Anhui Key Research and Development Projects, grant No. 1804h08020259 & 202104j07020040), and Natural Science Foundation of Anhui Province (grant No. 1908805MH260).

## Conflict of Interest

XZ, FW, LZ, and QO are employees of Nanjing Geneseeq Technology Inc., China.

The remaining authors declare that the research was conducted in the absence of any commercial or financial relationships that could be construed as a potential conflict of interest.

## Publisher’s Note

All claims expressed in this article are solely those of the authors and do not necessarily represent those of their affiliated organizations, or those of the publisher, the editors and the reviewers. Any product that may be evaluated in this article, or claim that may be made by its manufacturer, is not guaranteed or endorsed by the publisher.

## Abbreviations

BC, breast cancer; DEG, differentially expressed gene; ER, estrogen receptor; ET, endocrine therapy; FFPE, formalin-fixed paraffin-embedded; FISH, Fluorescence *in situ* hybridization; HER2, human epidermal growth factor receptor 2; HR, hormone receptor; IHC, immunohistochemistry; NAC, neoadjuvant chemotherapy; NGS, next-generation sequencing; non-pCR, non-pathologic complete response; pCR, pathologic complete response; PR, progesterone receptor; RNA-Seq, RNA sequencing; TNBC, triple-negative breast cancer; WES, whole-exome sequencing.
